# Ultrasound-guided brachial plexus hydrodissection combined with acupotomy release for the treatment of cervical spondylotic radiculopathy: a multicenter retrospective study

**DOI:** 10.3389/fnagi.2026.1846209

**Published:** 2026-07-13

**Authors:** Xiang Shang, Guo-Rui Luan, Yang-Chun Song, Fen Zhang, San-Bing Wu, Kai Geng, Hou-Shan Fang, Wei Wei, Zhen-Ya Wang, Han-Qing Zhao, Yong-Hui Yang, De-Hong Meng

**Affiliations:** 1 Anhui University of Chinese Medicine, Hefei, Anhui, China; 2 Shuguang Anhui Hospital Affiliated to Shanghai University of Traditional Chinese Medicine, Hefei, Anhui, China; 3 The Second Affiliated Hospital of Anhui University of Chinese Medicine, Hefei, Anhui, China; 4 Lu’an Traditional Chinese Medicine Hospital, Lu'an, Anhui, China; 5 Fanchang District Traditional Chinese Medicine Hospital, Wuhu, Anhui, China

**Keywords:** acupotomy, brachial plexus hydrodissection, cervical spondylotic radiculopathy, F-wave, ultrasound

## Abstract

**Introduction:**

The incidence of cervical spondylotic radiculopathy (CSR) remains high. Conventional conservative treatments have limited efficacy in relieving mechanical nerve root compression, while surgery is highly invasive. Both acupotomy release and ultrasound-guided brachial plexus hydrodissection are minimally invasive interventions for CSR. Nevertheless, high-level multicenter evidence-based medical data regarding the safety and efficacy of their combined application are still lacking.

**Methods:**

This multicenter retrospective study enrolled 264 patients with CSR treated at four traditional Chinese medicine hospitals in China between June 2024 and November 2025. The patients were divided into two groups according to the therapeutic regimen: the ultrasound-guided brachial plexus hydrodissection combined with acupotomy release group (HAG) and the ultrasound-guided acupotomy release group (AG). Patients in the AG underwent ultrasound-guided acupotomy release treatment, the target sites for acupotomy release included the C2/3, C4/5, and C6/7 zygapophyseal joints, the C2 spinous process, and the superior medial angles of the bilateral scapulae. Patients in the HAG additionally received ultrasound-guided brachial plexus hydrodissection via the scalene space. Neuroelectrophysiological indicators (F-wave occurrence rate and conduction velocity of the median, ulnar, and radial nerves) were assessed before treatment and at 8 weeks after treatment. he Neck Disability Index (NDI), 36-Item Short Form Health Survey (SF-36), Visual Analogue Scale (VAS), and Numeric Rating Scale (NRS) for the brachial plexus traction test were used to evaluate clinical efficacy at baseline and at 4, 8, and 16 weeks after treatment. Adverse events during treatment were simultaneously recorded.

**Results:**

A total of 212 patients with CSR received treatment in this study. During the follow-up period, 3 patients in the HAG were excluded due to self-administered medication and another 3 were excluded due to loss to follow-up; correspondingly, 5 patients in the AG were excluded due to self-administered medication and 2 were excluded due to loss to follow-up. Consequently, 199 patients with CSR were finally enrolled in this study, including 90 cases in the HAG and 109 cases in the AG. At 8 weeks after treatment, the F-wave occurrence rate and conduction velocity of the median, ulnar, and radial nerves were significantly higher in the HAG than in the AG (*p* < 0.05). The NDI, VAS, and NRS scores for the brachial plexus traction test in the HAG were markedly lower than those in the AG at all postoperative time points (*p* < 0.05), whereas the SF-36 score was notably higher (*p* < 0.05). Both groups of patients experienced transient postsurgical soreness and localized bruising after treatment, no major adverse events, including nerve injury or infection, were observed.

**Conclusion:**

Ultrasound-guided brachial plexus hydrodissection combined with acupotomy release is safe and effective in the treatment of CSR. Compared with acupotomy release alone, the combined therapy was associated with greater improvements in neurophysiological parameters, pain severity, upper-limb symptoms, cervical function, and quality of life, thus providing an optimized minimally invasive strategy for the clinical management of CSR.

## Introduction

Changes in modern working patterns, particularly prolonged desk-based activities, have contributed to a rising incidence of cervical spondylosis, which has been reported to reach 13.76% in recent epidemiological studies (Lv et al., 2018). Cervical spondylotic radiculopathy (CSR) is one of the most common subtypes of cervical spondylosis, accounting for approximately 24% of all patients with cervical spondylosis (Damm et al., 2023). Its primary pathological mechanism involves compression or irritation of the corresponding cervical nerve roots by herniated intervertebral discs, hypertrophic uncovertebral joints, or thickened ligaments, resulting in radiating symptoms such as pain, numbness, and weakness in the innervated regions (Peng et al., 2023). The manifestations in patients include neurological abnormalities, headache, blurred vision, dizziness, gastrointestinal discomfort, tinnitus, hypomnesia, and palpitations (Leung et al., 2023).

Current Western medical treatments for CSR mainly consist of oral nonsteroidal anti-inflammatory drugs and physical therapy. Although pharmacotherapy can alleviate periradicular inflammation, it fails to relieve the physical compression of nerve roots (Onda and Kimura, 2018). For most patients with moderate to severe CSR, monotherapy with medications or conventional physical therapy yields limited efficacy and is prone to recurrence. While surgical treatment provides definitive therapeutic effects, it is associated with high invasiveness, risks, and costs, leading to low patient acceptance and often insufficient surgical indications (Taso et al., 2025).

Traditional Chinese medicine has achieved remarkable clinical efficacy in the management of CSR (Yang et al., 2023). As a novel therapeutic instrument integrating the acupuncture needle of traditional Chinese medicine and the surgical scalpel, acupotomy can effectively release adhesions, contractures, and scars in the soft tissues of the neck and shoulder, thereby relieving neurocompression caused by soft tissues. It has been widely applied in the clinical treatment of CSR (Pu et al., 2023). In recent years, ultrasound-guided brachial plexus hydrodissection has emerged as an innovative minimally invasive interventional technique. By injecting fluids such as normal saline and local anesthetics around the compressed nerve roots, potentially improving the perineural microenvironment and reducing inflammatory irritation, thus rapidly alleviating pain and neurological symptoms. Nevertheless, although this combined therapy has been extensively used in clinical practice, high-quality multicenter clinical evidence for the systematic evaluation of its safety and efficacy is lacking, and there is an even greater shortage of high-level evidence-based medical data compared with conventional standard therapies.

This study aimed to analyze the safety and efficacy of ultrasound-guided brachial plexus hydrodissection combined with acupotomy release in the treatment of CSR through a multicenter retrospective study, so as to provide novel insights and valid evidence-based references for the clinical management of CSR.

## Materials and methods

### Study design

This was a multicenter retrospective study. To reduce therapeutic bias attributable to inter-operator variability, physicians at all centers received standardized training before treatment initiation, covering the manipulation techniques and target site selection for ultrasound-guided acupotomy release, as well as the injection sites and extent for ultrasound-guided brachial plexus hydrodissection.

### Patients

This study retrospectively analyzed patients with CSR who received ultrasound-guided brachial plexus hydrodissection combined with acupotomy release between June 2024 and November 2025 at The Second Affiliated Hospital of Anhui University of Chinese Medicine, Shuguang Anhui Hospital Affiliated to Shanghai University of Traditional Chinese Medicine, Lu’an Traditional Chinese Medicine Hospital, Fanchang District Traditional Chinese Medicine Hospital. Treatment allocation was determined according to the treatment regimen received at each participating center rather than MRI severity.

Inclusion criteria:(1) Patients diagnosed with CSR confirmed by clinical and imaging examinations;(2) Patients with upper limb sensory abnormalities lasting for more than 1 month;(3) Age older than 20 years;(4) No use of medications that may affect the study results within 2 weeks prior to treatment;(5) Complete clinical data available. Exclusion criteria:(1) Patients with a history of cervical spine surgery;(2) Upper limb sensory abnormalities not caused by CSR;(3) Patients with sensory abnormalities in both upper limbs;(4) Patients who unauthorizedly received treatments outside the study protocol during treatment or follow-up.

This study was conducted in strict accordance with the principles of the Declaration of Helsinki. Written informed consent was obtained from all patients before the corresponding treatment. The study protocol was approved by the Ethics Committee of Anhui Hospital of Shuguang Hospital Affiliated to Shanghai University of Traditional Chinese Medicine (Ethics Approval No.: 2025SGH-EAD-025).

### Intervention methods

During the treatment period, patients were advised to maintain their usual daily activities and did not participate in a standardized physiotherapy program.

Patients in HAG received ultrasound-guided brachial plexus hydrodissection combined with acupotomy release treatment.

**Figure 1:**
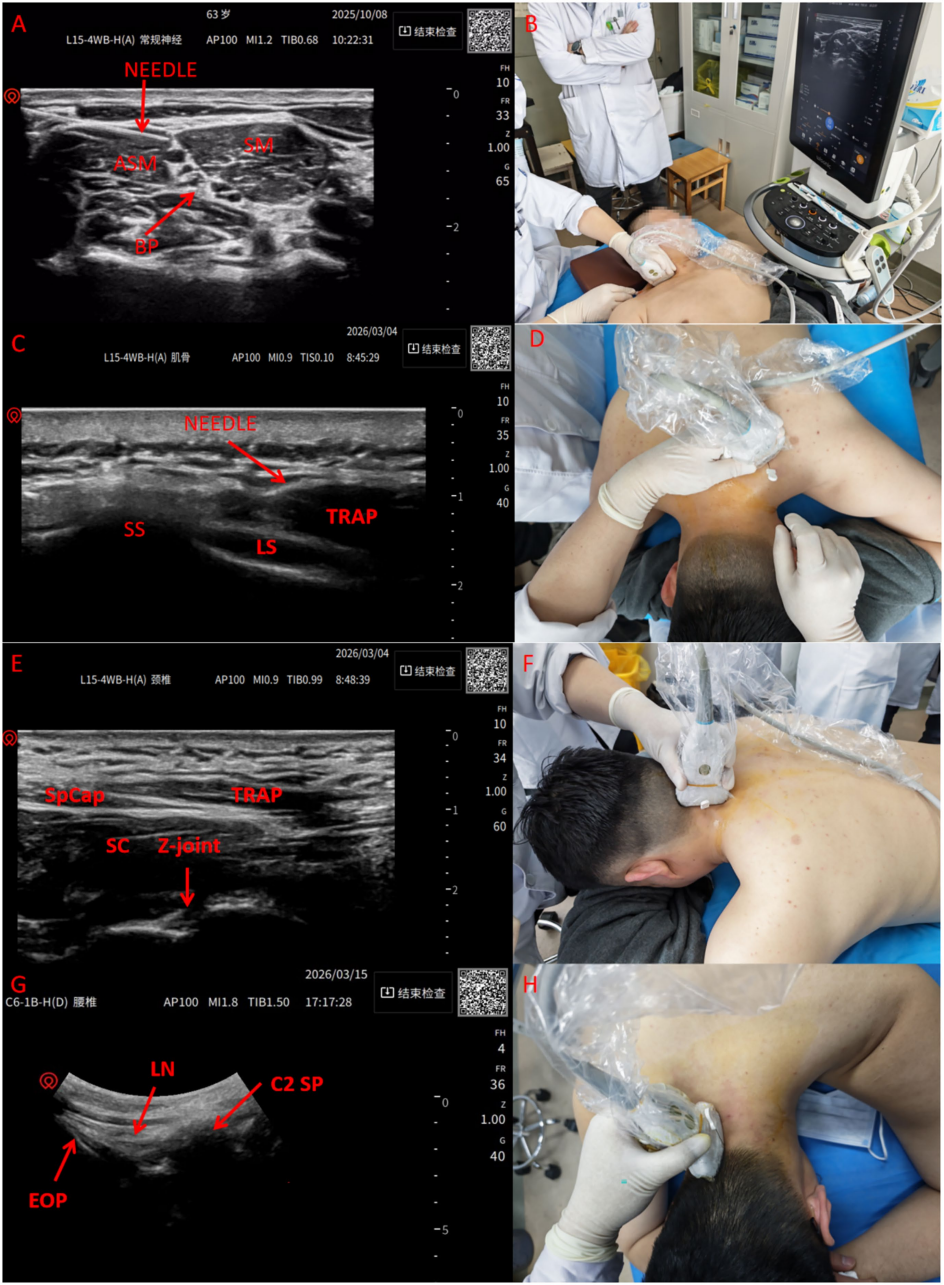
Ultrasound-guided brachial plexus hydrodissection combined with acupotomy release (**A, B**: Ultrasound images of ultrasound-guided brachial plexus hydrodissection. ASM, Anterior scalene muscle; SM, Middle scalene muscle; BP, Brachial plexus. **C, D**: Ultrasound images of ultrasound-guided acupotomy release for the levator scapulae. SS, Superior medial angle of the scapula; Trap, Trapezius; LS, Levator scapulae. **E, F**: Images of ultrasound-guided acupotomy release for the cervical zygapophyseal joints. SpCap, Splenius capitis; SC, Semispinalis capitis; Z-joint, Zygapophyseal joint. **G, H**: Images of ultrasound-guided acupotomy release for the C2 spinous process. LN, Ligamentum nuchae; C2 SP, Spinous process of C2; EOP, External occipital protuberance.).

The patient was first placed in the supine position with the head turned to the unaffected side to fully expose the neck skin, which was then subjected to routine disinfection on the affected side. Ultrasound examination was performed using a Wisonic-Navi device (License No.: 20122302) equipped with a linear array high-frequency probe (5–12 MHz) covered with a sterile protective sheath. The ultrasound probe was positioned above the clavicle on the affected side and oriented perpendicular to the scalene muscles. By moving the probe superiorly and inferiorly, the anterior and middle scalene muscles as well as the brachial plexus located between them could be visualized (Figures 1A,B). A sterile disposable puncture needle (0.50 × 60 mm) was advanced into the scalene space under ultrasound guidance, and 10 mL of 0.9% sodium chloride solution was injected into the space to perform hydrodissection of the brachial plexus (Video 1). The puncture site was compressed with sterile gauze after injection. The procedural endpoint of hydrodissection was visualization of circumferential fluid spread around the brachial plexus with clear separation between the neural elements and surrounding fascial tissues, without evidence of intraneural injection.

Following injection, the patient was instructed to assume the prone position with both hands overlapped under the forehead to fully expose the neck and shoulder skin. The target puncture sites included the bilateral C2/3, C4/5, and C6/7 zygapophyseal joints, the C2 spinous process, and the superior medial angles of the bilateral scapulae. After routine disinfection, a sterile disposable acupotomy (0.6 mm × 50 mm, Jiangsu Laozongyi Medical Devices Co., Ltd.) was used.

First, the zygapophyseal joints were released. Under real-time ultrasound guidance, the acupotomy was inserted perpendicularly to the skin with its blade aligned parallel to the longitudinal axis of the body, strictly following the four-step acupotomy insertion technique (localization, orientation, compression separation, and penetration). Care was taken to avoid vital structures such as peripheral blood vessels. The acupotomy was advanced until reaching the surface of the zygapophyseal joints, where three longitudinal lysis and transverse stripping maneuvers were performed. The blade was then rotated 90°, and three additional releases were conducted on the joint capsule before the acupotomy was withdrawn, followed by compression with sterile gauze (Video 2 and Figures 1E,F).

Second, the C2 spinous process was released using a low-frequency convex array probe (1–5 MHz). Under ultrasound guidance, the acupotomy was inserted perpendicularly to the skin with its blade aligned with the nuchal ligament, adhering strictly to the four-step insertion technique while avoiding adjacent vascular structures. The acupotomy was advanced to the bone surface of the C2 spinous process, where three longitudinal lysis and transverse stripping procedures were performed. The blade was rotated 90°, and three releases were performed on the superior and inferior nuchal ligaments before the acupotomy was removed and the site compressed with sterile gauze (Figures 1G,H).

Finally, the superior medial angles of the scapulae were released. Under ultrasound guidance, the acupotomy was inserted at a 45° angle to the skin with its blade oriented along the levator scapulae muscle, following the four-step insertion technique and avoiding vital periscapular structures. The acupotomy was advanced to the bone surface of the superior medial angle of the scapula, where three longitudinal lysis and transverse stripping maneuvers were conducted before withdrawal. Sterile gauze was applied for hemostasis (Figures 1C,D). Acupotomy release was administered once weekly for four consecutive sessions. Throughout the procedure, continuous ultrasound visualization was maintained to confirm blade position and avoid adjacent neurovascular structures.

Patients in AG received only ultrasound-guided acupotomy release treatment, with the same treatment sites and course as those in the HAG.

### Clinical data collection

Patients were followed up both online and offline to assess outcomes at baseline, 4, 8, and 16 weeks after treatment. Cervical function was evaluated using the Neck Disability Index (NDI) (Jones and Sterling, 2021); quality of life was assessed with the 36-Item Short Form Health Survey (SF-36) (Lins and Carvalho, 2016); cervical pain severity was measured using the Visual Analogue Scale (VAS) (Modarresi et al., 2021). The degree of upper limb sensory abnormality was assessed using the Numerical Rating Scale (NRS) for the affected-side brachial plexus traction test: the patient was seated with the head slightly turned toward the examined side. The examiner stood on the affected side, gently pushed the patient’s head with one hand, and held the wrist of the affected limb with the other hand to abduct the limb while applying relative traction. A score of 0 indicated no radiating numbness or pain, while a score of 10 indicated obvious radiating numbness or pain even without performing the brachial plexus traction test (Al-Khazali et al., 2024). The F-wave occurrence rate and conduction velocity of the ulnar, median, and radial nerves in the affected upper limb were measured using an electromyography/evoked potential system (YZB/USA6001-2013, Natus Neurology Incorporated, USA) at baseline and 8 weeks after treatment.

### Follow-up

Patients were followed up at 1 month after treatment completion, and adverse events occurring during the follow-up period were recorded, including nerve injury, muscle atrophy, infection, and other complications.

### Statistical analysis

Statistical analysis was performed using SPSS 26.0 (IBM Inc., Chicago, IL, USA). Normality of continuous variables was assessed using the Shapiro–Wilk test. Normally distributed variables were expressed as mean±standard deviation (SD) and analyzed using independent-samples or paired t-tests, as appropriate. Non-normally distributed variables were presented as *Median* (*P*_25_–*P*_75_) and analyzed using the Mann–Whitney U test or Wilcoxon signed-rank test. Categorical variables were compared using the chi-square test or Fisher’s exact test.

## Result

**Figure 2:**
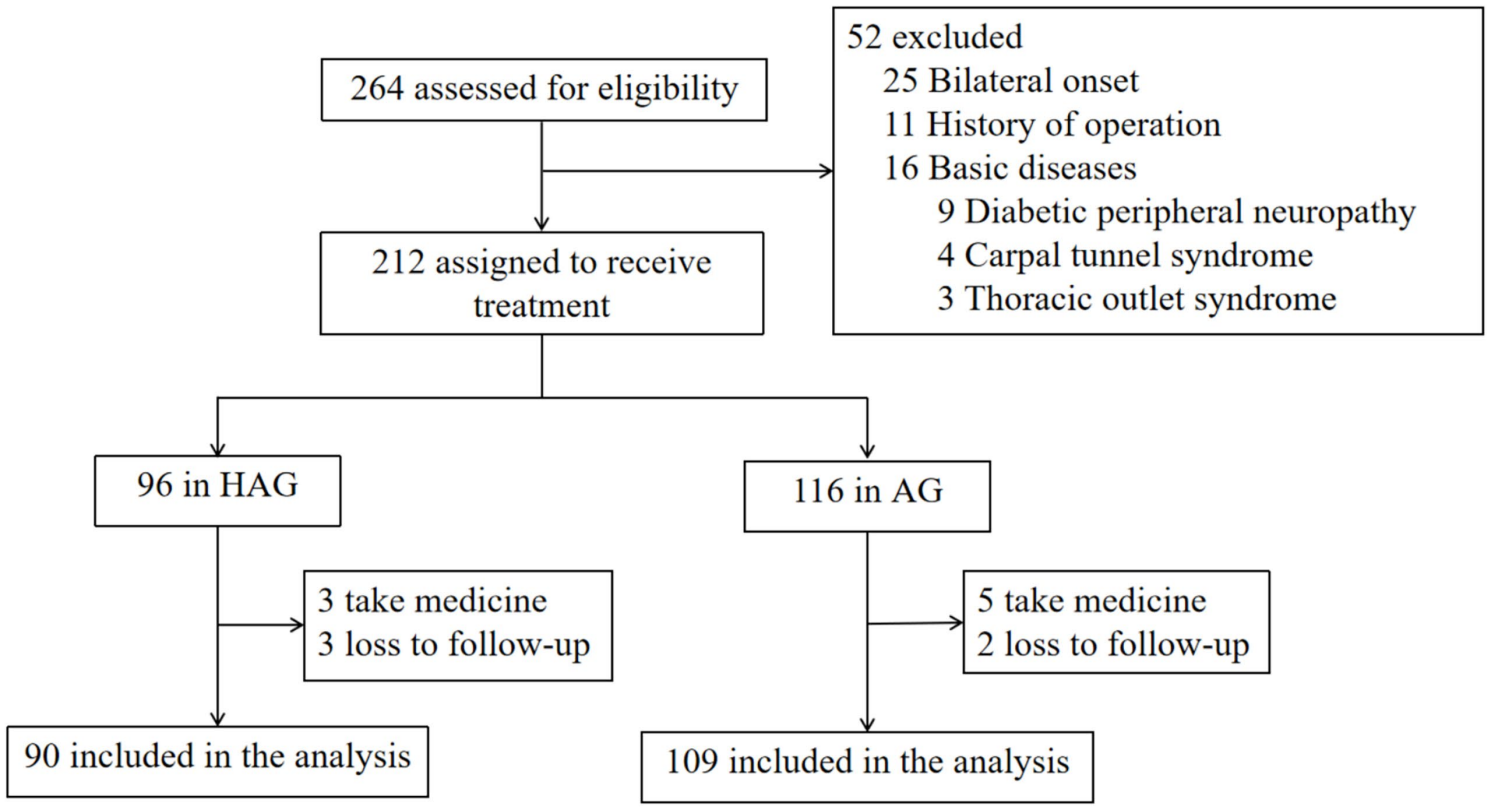
patient inclusion flow chart.

A total of 264 patients with CSR were initially enrolled in this study. Among them, 25 patients were excluded due to numbness in both upper limbs, 11 were excluded due to a history of cervical spine surgery, and 16 were excluded due to severe sensory abnormalities caused by other comorbidities (9 cases of diabetic peripheral neuropathy, 4 cases of carpal tunnel syndrome, and 3 cases of thoracic outlet syndrome). During the follow-up period, 3 patients in the HAG were excluded due to self-administered medication and an additional 3 were excluded due to loss to follow-up; meanwhile, 5 patients in the AG were excluded due to self-administered medication and 2 were excluded due to loss to follow-up. Consequently, 199 patients with CSR were finally included in the analysis, consisting of 90 cases in the HAG and 109 cases in the AG. The patient enrollment flowchart is shown in Figure 2. There were no statistically significant differences in baseline characteristics between the two groups, including age, disease duration, and neuroelectrophysiological results (*p* > 0.05), as presented in Table 1.

**Table 1:** Comparison of baseline data between 2 groups.

Characteristics	HAG (*n* = 90)	AG (*n* = 109)	*p* value
Gender, *n* (%)			
Male	36 (40.00)	44 (40.37)	0.958
Female	54 (60.00)	65 (59.63)	
Age (mean ± SD, years)	48.20 ± 11.13	47.40 ± 11.68	0.624
Course of disease (mean ± SD, months)	6.54 ± 2.19	7.26 ± 1.97	0.424
BMI (mean ± SD, kg/m^2^)	26.35 ± 2.08	26.29 ± 1.96	0.602
Affected side, *n* (%)			
Right	51 (56.67)	60 (55.05)	0.819
Left	39 (43.33)	49 (44.95)	
Hand dominance, *n* (%)			
Left	5 (5.56)	11 (10.09)	0.241
Right	85 (94.44)	98 (89.91)	
Engage in manual labor, *n* (%)			
Yes	32 (35.56)	31 (28.44)	0.283
No	58 (64.44)	78 (71.56)	
Comorbidities, *n* (%)			
Diabetes	13 (14.44)	16 (14.68)	0.963
Hypertension	15 (16.66)	19 (17.43)	0.887
Stroke	2 (2.22)	3 (2.75)	0.812
F-wave occurrence, *n* (%)			
Median nerve	56 (62.22)	67 (61.47)	0.913
Ulnar nerve	65 (72.22)	77 (70.64)	0.806
Radial nerve	57 (63.33)	73 (66.97)	0.591
F-wave velocity (mean ± SD, m/s)			
Median nerve	42.37 ± 6.71	40.93 ± 5.72	0.104
Ulnar nerve	55.31 ± 7.81	54.06 ± 7.39	0.248
Radial nerve	40.47 ± 5.03	41.75 ± 5.93	0.106

HAG, Ultrasound-guided brachial plexus hydrodissection combined with acupotomy release group; AG, Ultrasound-guided acupotomy release group; BMI, Body mass index; SD, Standard deviation.

### Neuroelectrophysiological

**Figure 3:**
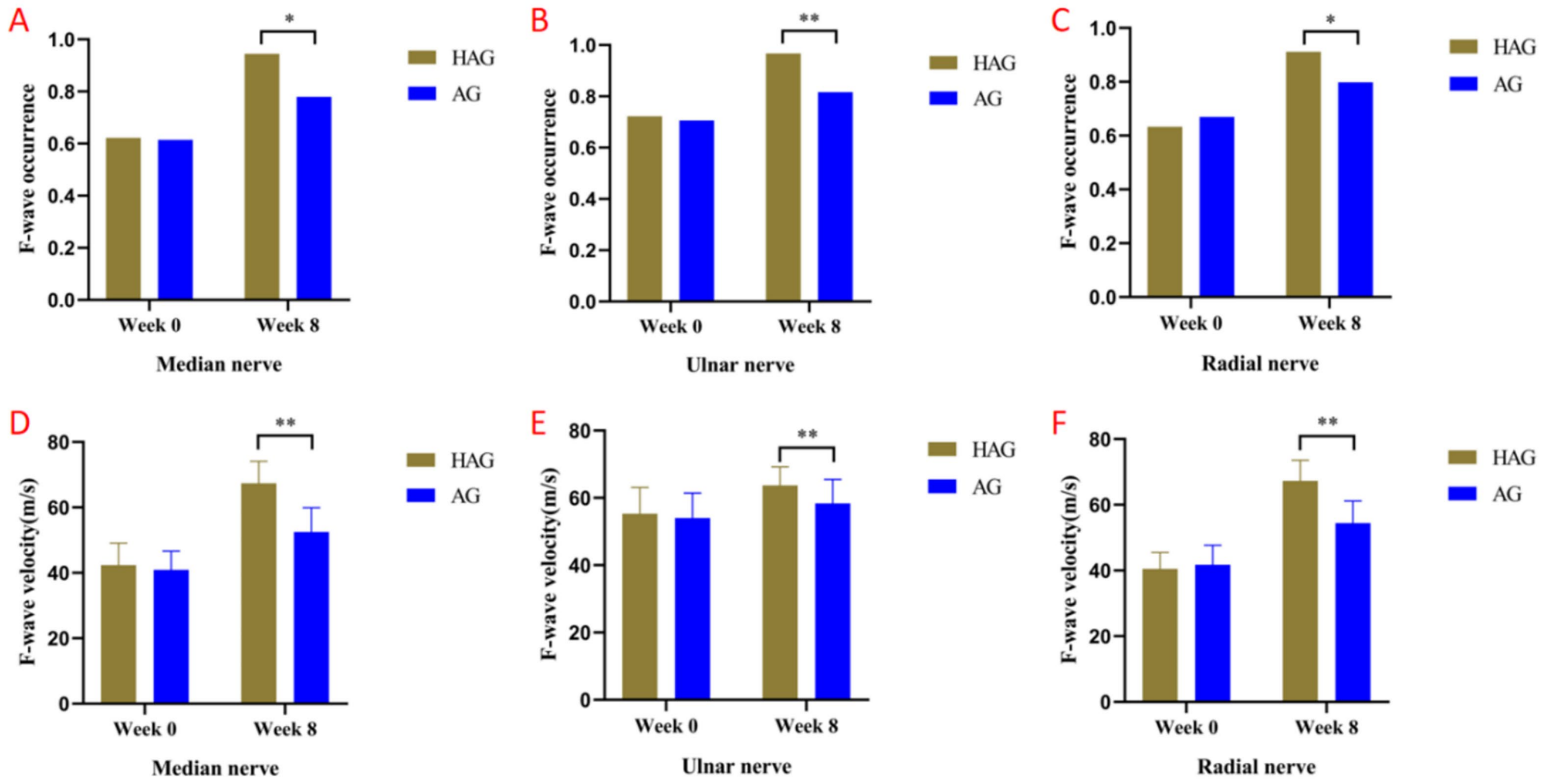
Results of neurophysiological indexes in 2 groups (^*^p < 0.05, ^**^p < 0.001). **(A–C)** show the occurrence rates of F-wave for Median nerve, Ulnar nerve, and Radial nerve; **(D–F)** show the F-wave velocities for Median nerve, Ulnar nerve, and Radial nerve.

To investigate the effect of ultrasound-guided brachial plexus hydrodissection combined with acupotomy release on the F-wave occurrence rate in the upper limbs of patients with cervical spondylotic radiculopathy, neuroelectrophysiological examinations were performed in both groups at week 0 and week 8. The results showed no significant between-group differences in the F-wave occurrence rate of the median nerve, ulnar nerve, and radial nerve at week 0 (*p* > 0.05). After treatment, the F-wave occurrence rate was significantly increased in both groups, and the values in the HAG were markedly higher than those in the AG at week 8 (*p* < 0.05). The results are presented in Table 2 and Figures 3A–C.

**Table 2:** Comparison of the occurrence rate of F-wave at different times between 2 groups (*n*, %).

Nerve	Time	HAG (*n* = 90)	AG (*n* = 109)	*p* value
Median nerve	Week 0	56 (62.22)	67 (61.47)	0.913
	Week 8	85 (94.44)^*^	85 (77.98)	0.0011
Ulnar nerve	Week 0	65 (72.22)	77 (70.64)	0.806
	Week 8	87 (96.67)^**^	89 (81.65)	<0.001
Radial nerve	Week 0	57 (63.33)	73 (66.97)	0.591
	Week 8	82 (91.11)^*^	87 (79.82)	0.027

HAG, Ultrasound-guided brachial plexus hydrodissection combined with acupotomy release group; AG, Ultrasound-guided acupotomy release group. Compared with AG, ^*^p < 0.05, ^**^p < 0.001.

To further investigate the effect of ultrasound-guided brachial plexus hydrodissection combined with acupotomy release on the upper limb F-wave velocity in patients with cervical spondylotic radiculopathy, we recorded and compared the F-wave velocity of all patients in whom F-waves were detectable at week 0. The results demonstrated no significant between-group differences in the F-wave velocity of the median nerve, ulnar nerve, and radial nerve at week 0 (*p* > 0.05). Following treatment, the F-wave velocity was significantly elevated in both groups, and the F-wave velocity in the HAG was markedly higher than that in the AG at week 8 (*p* < 0.05). The results are presented in Table 3 and Figures 3D–F.

**Table 3:** Comparison of the F-wave velocity at different times between 2 groups (mean ± SD, m/s).

Nerve	Time	HAG (*n* = 90)	AG (*n* = 109)	*p* value
Median nerve	Week 0	42.37 ± 6.71	40.93 ± 5.72	0.104
	Week 8	67.36 ± 6.77^**^	52.51 ± 7.38	<0.001
Ulnar nerve	Week 0	55.31 ± 7.81	54.06 ± 7.39	0.248
	Week 8	63.69 ± 5.51^**^	58.34 ± 7.18	<0.001
Radial nerve	Week 0	40.47 ± 5.03	41.75 ± 5.93	0.106
	Week 8	67.28 ± 6.29^**^	54.40 ± 6.79	<0.001

HAG, Ultrasound-guided brachial plexus hydrodissection combined with acupotomy release group; AG, Ultrasound-guided acupotomy release group; SD, Standard deviation. Compared with AG, ^**^p < 0.001.

### Neck disability index score

**Figure 4:**
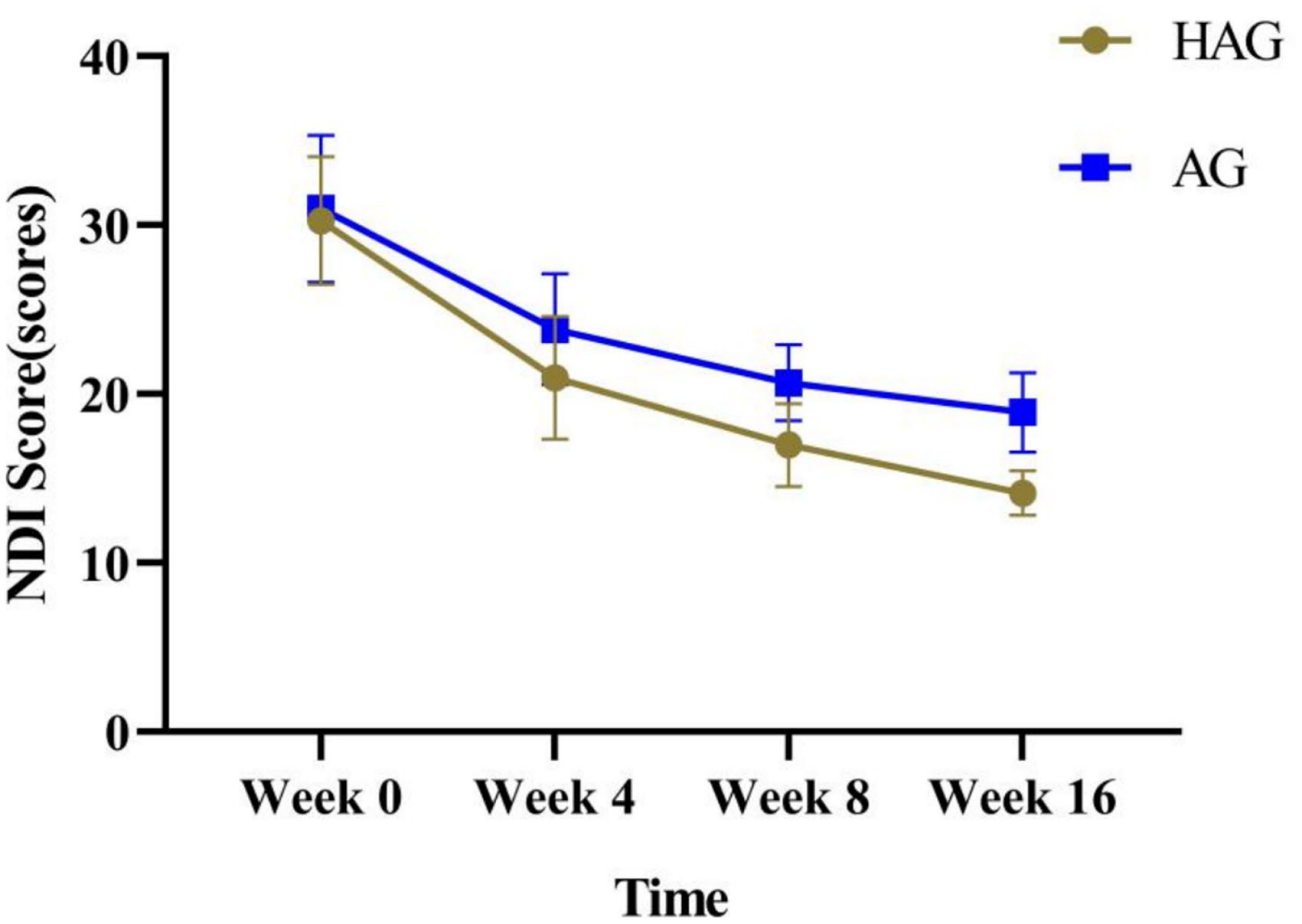
Comparison of NDI scores between 2 groups.

No significant difference was observed in the NDI score between the two groups before treatment (*p* > 0.05). After treatment, the NDI scores decreased in both groups, and the NDI scores in the HAG were lower than those in the AG at all post-treatment time points (*p* < 0.05). The results are presented in Table 4 and Figure 4.

**Table 4:** Comparison of NDI scores at different time points after treatment between 2 groups (mean ± SD, scores).

Group	Week 0	Week 4	Week 8	Week 16
HAG	30.26 ± 3.78	20.94 ± 3.64^**^	16.97 ± 2.46^**^	14.13 ± 1.32^**^
AG	30.97 ± 4.36	23.83 ± 3.29	20.65 ± 2.25	18.91 ± 2.35
*p* value	0.226	<0.001	<0.001	<0.001

NDI, Neck Disability Index; HAG, Ultrasound-guided brachial plexus hydrodissection combined with acupotomy release group; AG, Ultrasound-guided acupotomy release group; SD, Standard deviation. Compared with AG, ^**^p < 0.001.

### The short-From-36 health survey score

**Figure 5:**
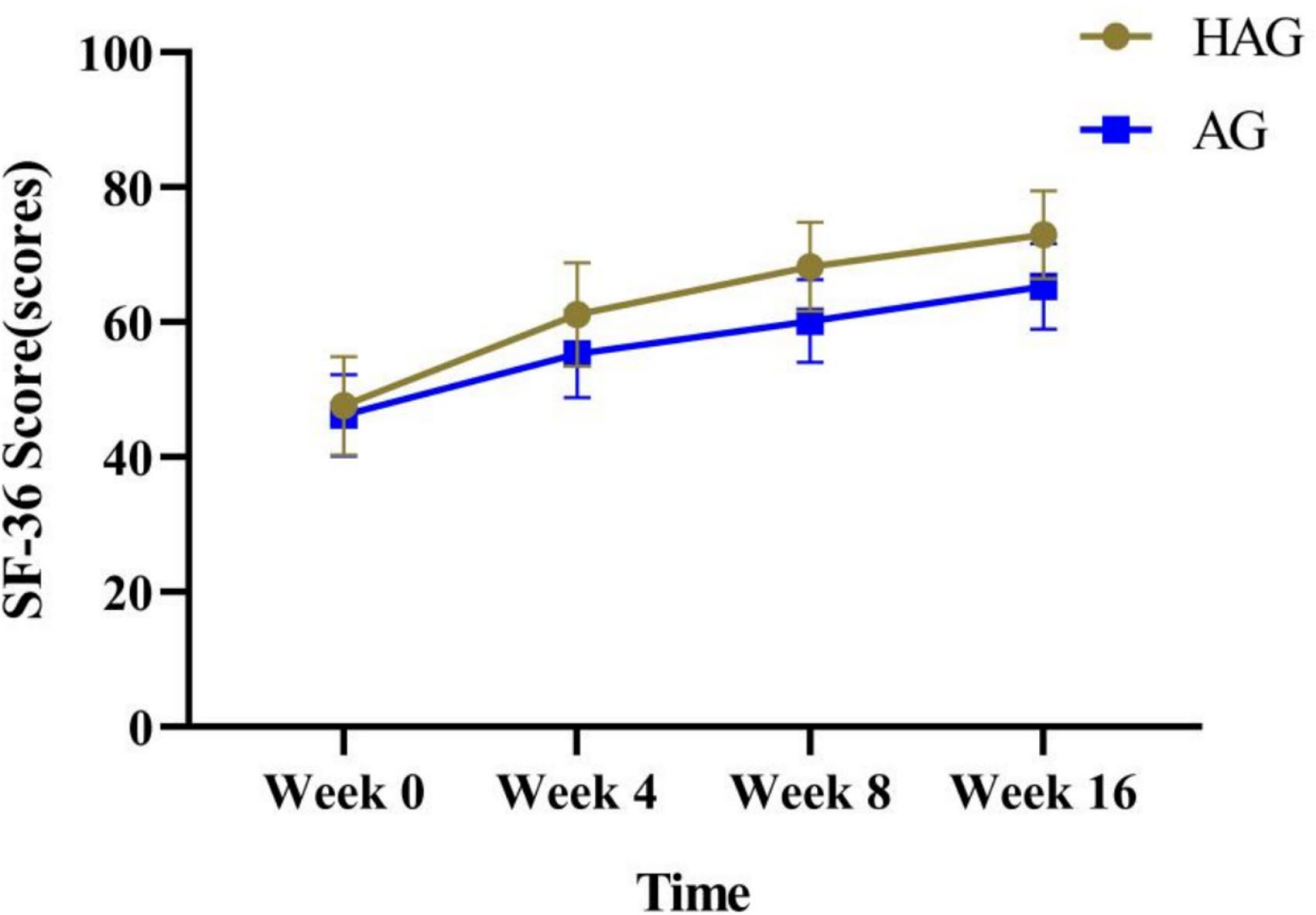
Comparison of SF-36 scores between 2 groups.

No significant difference in SF-36 scores was observed between the two groups before treatment (*p* > 0.05). Following treatment, SF-36 scores increased in both groups, and the scores in the HAG were higher than those in the AG at all post-treatment time points (*p* < 0.05). The results are presented in Table 5 and Figure 5.

**Table 5:** Comparison of SF-36 scores at different time points after treatment between 2 groups (mean ± SD, scores).

Group	Week 0	Week 4	Week 8	Week 16
HAG	47.62 ± 7.29	61.12 ± 7.65^**^	68.21 ± 6.55^**^	72.93 ± 6.49^**^
AG	46.17 ± 6.04	55.27 ± 6.47	60.14 ± 6.14	65.27 ± 6.35
*p* value	0.126	<0.001	<0.001	<0.001

SF-36, The Short-From-36 Health Survey; HAG, Ultrasound-guided brachial plexus hydrodissection combined with acupotomy release group; AG, Ultrasound-guided acupotomy release group; SD, Standard deviation. Compared with AG, ^**^p < 0.001.

### Visual analog scale score

**Figure 6:**
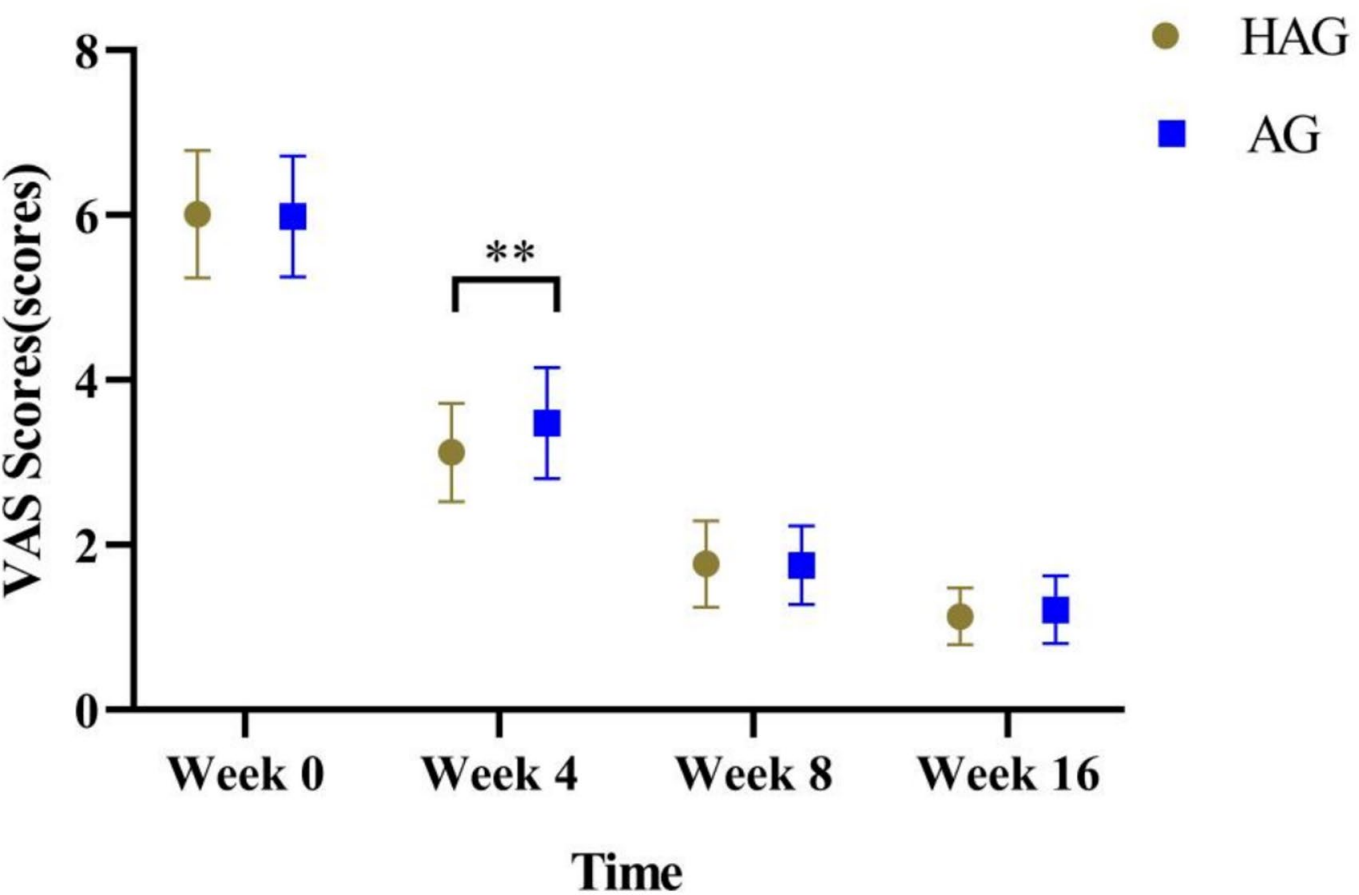
Comparison of VAS scores between 2 groups (^**^p < 0.001).

No significant difference in VAS scores was observed between the two groups before treatment (*p* > 0.05). After treatment, VAS scores decreased in both groups, and the scores in the HAG were lower than those in the AG at all post-treatment time points (*p* < 0.05). The results are presented in Table 6 and Figure 6.

**Table 6:** Comparison of VAS scores at different time points after treatment between 2 groups [*M* (*P*25, *P*75), scores].

Group	Week 0	Week 4	Week 8	Week 16
HAG	6 (6, 6)	3 (3, 3)^**^	2 (1, 2)	1 (1, 1)
AG	6 (6, 6)	3 (3, 4)	2 (1, 2)	1 (1, 1)
*p* value	0.204	<0.001	0.462	0.061

VAS, Visual Analogue Scale; HAG, Ultrasound-guided brachial plexus hydrodissection combined with acupotomy release group; AG, Ultrasound-guided acupotomy release group. Compared with AG, ^**^p < 0.001.

### Numerical rating scale score for brachial plexus traction test

**Figure 7:**
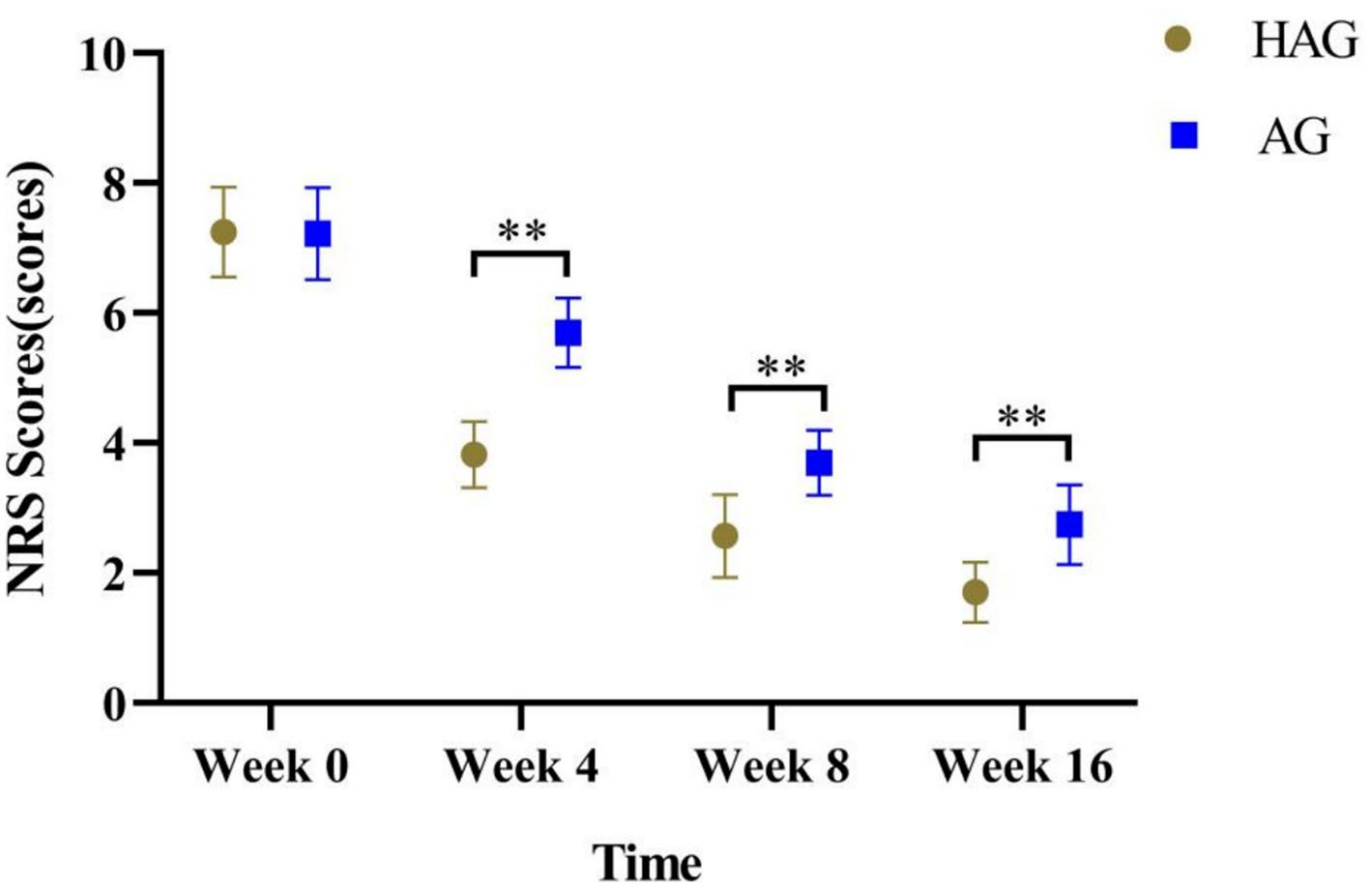
Comparison of NRS scores between 2 groups (^**^p < 0.001).

No significant difference was observed in the NRS score of the brachial plexus traction test between the two groups before treatment (*p* > 0.05). After treatment, the NRS scores of the brachial plexus traction test decreased in both groups, and the scores in the HAG were lower than those in the AG at all post-treatment time points (*p* < 0.05). The results are presented in Table 7 and Figure 7.

**Table 7:** Comparison of NRS scores for brachial plexus traction test at different time points after treatment between 2 groups [*M* (*P*25, *P*75), scores].

Group	Week 0	Week 4	Week 8	Week 16
HAG	7 (7, 8)	4 (4, 4)^**^	3 (2, 3)^**^	2 (1, 2)^**^
AG	7 (7, 8)	6 (5, 6)	4 (3, 4)	3 (2, 3)
*p* value	0.529	<0.001	<0.001	<0.001

NRS, Numerical Rating Scale; HAG, Ultrasound-guided brachial plexus hydrodissection combined with acupotomy release group; AG, Ultrasound-guided acupotomy release group. Compared with AG, ^**^p < 0.001.

### Adverse events

Minor adverse events including transient postprocedural soreness, localized bruising, and temporary symptom aggravation were also reviewed. Most patients in HAG and AG reported experienced transient postprocedural soreness after Acupotomy release, 14 patients in HAG developed localized bruising after treatment, 20 patients in AG developed localized bruising after treatment, all lesions resolved spontaneously within 1 week. No major complications, including nerve injury, infection, persistent neurological deficit, or hospitalization, were identified.

## Discussion

The cervical spine serves as a pivotal hub connecting the skull and trunk. It not only bears the entire weight of the head but also enables multi-directional movements of the head and neck. Its anatomical structure encompasses numerous vital tissues including blood vessels, nerves, and the spinal cord, conferring unique complexity and functionality. The cervical vertebral bodies are relatively small, whereas the intervertebral foramina are comparatively large. After emerging from the spinal cord, the cervical nerve roots traverse the intervertebral foramina formed by the uncovertebral joints, intervertebral discs, zygapophyseal joints, and ligamenta flava, and extend distally to innervate the corresponding muscles (Poudel et al., 2025).

Pathological alterations in any anatomical structure within this region, including herniated intervertebral discs, osteophyte formation of the uncovertebral or zygapophyseal joints, and hypertrophy of the ligamenta flava, may result in relative or absolute stenosis of the intervertebral foramina, subsequently leading to compression and adhesion of the traversing nerve roots (Caballero et al., 2025). Such compression involves not only mechanical entrapment but is also frequently accompanied by local aseptic inflammation and circulatory disturbance, which further irritate the epineurium and induce edema of the nerve roots and impaired neural conduction. Clinically, this manifests as a series of radicular symptoms such as pain, numbness, sensory abnormalities, and decreased muscle strength distributed along the innervated regions of the affected nerve roots (Li and Cao, 2025; Kang et al., 2020).

At present, clinical therapeutic strategies for cervical spondylotic radiculopathy have become increasingly diversified, encompassing conservative treatments such as oral nonsteroidal anti-inflammatory drugs, neurotrophic medications, and physical therapy (Hirai et al., 2025; Yu et al., 2025; Núñez de Arenas-Arroyo et al., 2025). Although these interventions can alleviate pain and local inflammation to a certain extent, their efficacy in relieving mechanical compression of nerve roots is limited, and symptoms tend to recur repeatedly, especially in patients with a prolonged disease course and severe adhesions (Tayboga et al., 2025). Surgical treatment can directly decompress the affected structures; however, its high invasiveness, substantial costs, and potential surgical risks restrict patient acceptance (Liawrungrueang et al., 2025). Therefore, contemporary management of CSR increasingly emphasizes precision-based and minimally traumatic interventions. In clinical practice, treatment selection should be individualized according to the predominant pain mechanism, symptom severity, functional impairment, imaging findings, and response to conservative therapy. From a precision pain-management perspective, clinical decision-making should follow a stepwise treatment algorithm. The initial stage focuses on functional assessment and rehabilitation-based management aimed at correcting postural imbalance, muscular dysfunction, and altered cervicoscapular biomechanics. In patients with persistent symptoms or predominant myofascial pain components, ultrasound-guided dry needling and other minimally invasive neuromodulatory techniques may be considered as intermediate interventions. For selected patients with refractory symptoms, suspected perineural adhesions, fibrosis, or dynamic neural entrapment, escalation to ultrasound-guided hydrodissection and/or acupotomy release may be appropriate (Table 8). This hierarchical strategy emphasizes individualized treatment selection rather than a uniform superiority of any single intervention. Importantly, these techniques should not be interpreted as competing interventions. Instead, they represent complementary approaches targeting different anatomical layers, including myofascial structures, periarticular tissues, and neural interfaces.

**Table 8:** Comparison with DN-US and other minimally invasive techniques.

Aspect	DN-US	Acupotomy	Hydrodissection
Primary target	Myofascial trigger points, dysfunctional muscle chains	Fibrotic tissue, fascial adhesions, periarticular structures	Neural interface, perineural tissue planes
Main mechanism	Neuromuscular modulation	Mechanical release	Fluid-mediated separation
Tissue trauma	Lower	Higher	Intermediate
Typical clinical role	Functional pain phenotypes	Refractory structural dysfunction	Dynamic nerve entrapment and neural adhesions

As a representative minimally invasive technique in traditional Chinese medicine, acupotomy therapy not only integrates the key function of surgical incision and release but also avoids extensive incisions associated with conventional scalpels due to its acupuncture needle-like appearance (Guo-Rui et al., 2025). Acupotomy has demonstrated unique advantages in the treatment of CSR by releasing adhesions and scars in the soft tissues of the neck and shoulder (Lijian et al., 2025). Nevertheless, acupotomy mainly acts on the bone surface and fascial layers, and sometimes fails to achieve precise and full-range release for extensive adhesions formed between nerve roots and surrounding soft tissues. Ultrasound-guided brachial plexus hydrodissection can bluntly dissect adhesions between nerves and fasciae via hydraulic pressure, featuring a wide dissection range and a relatively gentle procedure without damaging nerves or blood vessels. Combined with ultrasound-guided acupotomy release, it enables multi-target and comprehensive intervention for CSR.

The results of the present study demonstrated that ultrasound-guided brachial plexus hydrodissection combined with acupotomy release was significantly superior to ultrasound-guided acupotomy release alone in improving neurological function, alleviating pain, and enhancing quality of life in patients with CSR. This superiority was first reflected in the remarkable improvement of neuroelectrophysiological indicators. The F-wave is a sensitive indicator reflecting the conduction function of the proximal segment of motor nerves. When nerve roots are compressed, the F-wave occurrence rate decreases, with prolonged latency or slowed conduction velocity (Lin et al., 2013). The results of this study showed that the F-wave occurrence rate and conduction velocity of the median, ulnar, and radial nerves on the affected side were significantly higher in the HAG than in the AG at 4, 8, and 16 weeks after treatment. These findings suggest that the combined intervention may improve proximal nerve function through multiple mechanisms, including reduction of neural mechanosensitivity, improvement of the perineural microenvironment, modulation of nociceptive input, and possible reduction of soft tissue-related neural irritation.

Zygapophyseal joints are synovial joints whose synovial layers are rich in nerve endings. When zygapophyseal joints are misaligned or subjected to abnormal stress, synovial entrapment may occur, accompanied by increased intra-articular pressure, which stimulates nerve endings in the synovium and causes severe pain (Won and Kim, 2025). Among them, the zygapophyseal joints at the C4/5 and C6/7 levels are regions where cervical flexion, extension, and rotational stresses are relatively concentrated, and are also frequent sites of cervical disc herniation and osteoproliferation. Relaxation of the joint capsule or degenerative changes of the intervertebral disc leading to narrowing of the intervertebral space can directly compress the C5-7 nerve roots. Acupotomy release of the zygapophyseal joint capsule may reduce capsular tension and potentially contribute to improved foraminal mechanics and reduced neural irritation. Although the incidence of C2/3 intervertebral disc herniation is lower than that of the lower cervical segments, zygapophyseal joint disorders in this region are often an important source of cervicogenic headache and cervical tension (Govind and Bogduk, 2022). Acupotomy release of the C2/3 zygapophyseal joints may contribute to improved biomechanical coordination of the upper cervical region and reduce the burden on the lower cervical spine caused by compensatory tension of the upper cervical spine.

The C2 spinous process is the main attachment site for numerous deep cervical muscles, such as the rectus capitis posterior major muscle and the obliquus capitis superior muscle, as well as a key insertion area of the ligamentum nuchae. In patients with CSR, deep cervical muscles are often in a state of spasm due to pain stimulation and postural compensation. As a stress center, the C2 spinous process is usually a high-stress point for soft tissue contracture and adhesion (Sakaura et al., 2008). Release of the C2 spinous process may help reduce excessive tension in the upper cervical spine and may facilitate reduction of excessive muscular tension by adjusting the suboccipital muscles, creating favorable conditions for the recovery of the lower cervical spine.

The superior medial angle of the scapula is the insertion site of the levator scapulae and rhomboid muscles. The cervical spine is located on the mobile thorax, and the scapula is suspended on the thorax via muscles; thus, scapular stability is the foundation of cervical stability. If only the cervical spine is released without scapular adjustment, symptoms tend to recur easily after treatment. Acupotomy release of the superior medial angle of the scapula can reduce distal tension of the levator scapulae muscle, which may be associated with changes in cervicoscapular functional biomechanics.

The pathological process of cervical spondylotic radiculopathy is not limited to compression within the bony intervertebral foramen. After exiting the intervertebral foramen, the nerve root passes through the space between the anterior and middle scalene muscles. Long-term chronic inflammatory stimulation can lead to adhesions between the fascia of the scalene space and the nerve trunk, forming a second entrapment site of the nerve (Lucas et al., 2020), which is also an important reason why some patients still suffer from persistent upper limb numbness despite relief of neck pain after treatment. However, for CSR patients with multi-level disc herniation in clinical practice, it is difficult for acupotomy to achieve precise release of each compressed nerve root. By targeting the brachial plexus trunk in the scalene space, hydrodissection uses fluid hydrostatic pressure to gently and atraumatically separate the nerve trunk from the surrounding adherent muscles and connective tissues, thereby relieving dynamic nerve entrapment. The injected fluid can dilute locally accumulated inflammatory factors and improve microcirculatory blood flow inside and outside the nerve through mild stretching of the epineurium, accelerating edema resolution and providing a clean microenvironment for the repair of damaged nerves (Buntragulpoontawee et al., 2021; Serrano et al., 2025). For nerve channel stenosis caused by muscle spasm, fluid injection may transiently expand the scalene space and reduce neural mechanosensitivity, although the exact mechanisms require further investigation.

In summary, acupotomy focuses on relieving entrapment and mechanical imbalance at the intervertebral foramen region and bony surface stress points, whereas hydrodissection prioritizes releasing soft tissue adhesions of the nerve trunk within soft tissue pathways. The combination of the two modalities addresses both bony and soft tissue pathologies, potentially addressing multiple pathological processes including neural irritation, myofascial dysfunction, altered biomechanics, and perineural adhesion. These complementary mechanisms may partly explain in various clinical scores. In the present study, improvements in NDI, SF-36, VAS, and NRS scores of the brachial plexus traction test were significantly better in the HAG than in the AG at 4, 8, and 16 weeks after treatment. Notably, although there was no statistically significant difference in VAS scores between the two groups at 8 and 16 weeks, the NRS scores of the brachial plexus traction test remained consistently superior in the HAG. This suggests that the combined therapy has a more durable advantage in improving upper limb numbness, while acupotomy alone has already exhibited significant clinical efficacy in relieving neck pain.

Degenerative changes of the cervical spine increase with aging and frequently contribute to chronic pain, sensory dysfunction, and impaired quality of life. Because older adults are often less suitable candidates for surgical intervention due to comorbidities and perioperative risks, minimally invasive image-guided interventions may represent an important therapeutic option in this population. Future studies should specifically evaluate the effectiveness of this strategy in older patients with degenerative cervical disorders.

As a multicenter retrospective study, this study reduced operator bias from a single center to a certain extent and ensured sample homogeneity through strict inclusion and exclusion criteria. This study has several limitations. First, as a retrospective multicenter analysis, it is susceptible to selection bias and residual confounding despite comparable baseline characteristics between groups. MRI findings and severity of foraminal stenosis were heterogeneous among patients and were not used as stratification variables in this retrospective analysis. In addition, no standardized physiotherapy or rehabilitation program was implemented across centers. As rehabilitation interventions may influence clinical outcomes, this factor could not be fully controlled in the present study. Second, the follow-up period was limited to 16 weeks, preventing assessment of long-term outcomes and recurrence rates. Third, ultrasound was primarily used for procedural guidance rather than quantitative outcome assessment. Future prospective randomized controlled trials with longer follow-up and objective imaging biomarkers are warranted to validate the present findings. In addition, repeated measurements were analyzed using time-point comparisons rather than mixed-effects longitudinal models, which should be considered in future studies. Future studies should incorporate dynamic ultrasound evaluation, elastography, quantitative assessment of nerve mobility and fascial sliding, and other imaging biomarkers to validate the biological mechanisms proposed in the present study. These limitations should be carefully considered when interpreting the results and highlight the need for prospective, controlled studies.

## Conclusion

This multicenter retrospective analysis suggests that ultrasound-guided brachial plexus hydrodissection combined with acupotomy release appears to be a safe and potentially effective minimally invasive treatment option for patients with cervical spondylotic radiculopathy. Compared with ultrasound-guided acupotomy release alone, the combined intervention was associated with greater improvements in neurophysiological parameters, pain severity, upper-limb symptoms, cervical function, and quality of life.

The observed benefits may be explained by several potential mechanisms, including modulation of neural mechanosensitivity and the perineural microenvironment, reduction of soft tissue-related neural irritation, and possible improvements in cervicoscapular biomechanics. However, the present findings should not be interpreted as direct evidence of structural decompression, and further studies incorporating objective imaging and functional assessments are needed to clarify the underlying mechanisms.

Given the growing burden of degenerative cervical spine disorders in aging populations, this minimally invasive approach may provide a valuable therapeutic option for patients who are unsuitable for surgery or who seek alternatives to more invasive procedures. Its potential to improve pain, neurological function, and quality of life while avoiding major surgery may be particularly relevant for older adults with cervical degenerative diseases.

## Data Availability

The raw data supporting the conclusions of this article will be made available by the authors, without undue reservation.
